# Supplementary material for: The Role of Nicotinamide Mononucleotide Supplementation in Psoriasis Treatment
Source: Antioxidants (Basel). 2024 Feb 1;13(2):186. doi: 10.3390/antiox13020186 (PMC10886094; doi:10.3390/antiox13020186)
Supplement: Supplementary file 1 [file antioxidants-13-00186-s001.zip › supplementary figure.pdf]

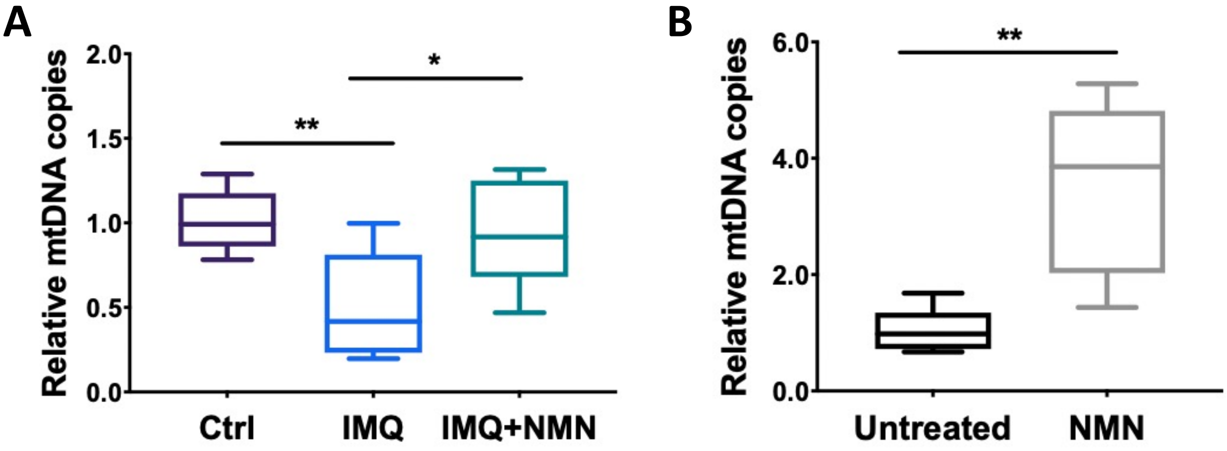

Supplementary Figure S1. mtDNA copies were increased under NMN treatment.

(A) Relative mtDNA copies in Ctrl, IMQ, IMQ+NMN.

(B) Relative mtDNA copies in Untreated, NMN.

Data are mean ± SEM, n=6 per group, * *p* < 0.05; ** *p* < 0.01; *** *p* < 0.001; ns, no significance.
