# Supplementary material for: The Role of Nicotinamide Mononucleotide Supplementation in Psoriasis Treatment
Source: Antioxidants (Basel). 2024 Feb 1;13(2):186. doi: 10.3390/antiox13020186 (PMC10886094; doi:10.3390/antiox13020186)
Supplement: Supplementary file 1 [file antioxidants-13-00186-s001.zip › Supplementary Tables.pdf]

Supplementary Table S1. Primers (*homo species*) used for RT-PCR

Genes	Forward	Reverse
IL-6	AAGCCAGAGCTGTGCAGATGAGTA	AAGCCAGAGCTGTGCAGATGAGTA
IL-8	GTCCTTGTTCCACTG TGCCT	GCTTCCACATGTCCTCACAA
IL-1α	TGGCTCA TTTTCCCTCAAAAGTTG	AGAAATCGTGAAATCCGA AGTCAAG
IL-1β	CCAGGGACAGGATATGG AGCA	CCAGGGACAGGATATGG AGCA
TNF-α	TCCTTCAGACACCCTCAACC	TCCTTCAGACACCCTCAACC
D-LOOP1	GTACTCCCGATTGAAGCCCC	CGATGGGCATGAAACTGTGG
D-LOOP2	GCTGTCCCCACATTAGGCTT	GCTCTAGAGGGGGTAGAGGG
D-LOOP3	CCGGGGGTATACTACGGTCA	GTGGGCTCTAGAGGGGGTAG
GAPDH	ACAACTTTGGTATCGTGGAAGG	GCCATCACGCCACAGTTTC
SIRT1	TAGCCTTGTCAGATAAGGAAGGA	TAGCCTTGTCAGATAAGGAAGGA

Supplementary Table S2. Primers (*mus musculus*) used for RT-PCR

Genes	Forward	Reverse
Il-6	CACTTCACAAGTCGGAGGCT	GCCACTCCTTCTGTGACTCC
Il-1α	CGAAGACTACGTTCTGCCATT	GACGTTTCAGAGGTTCTCAGAG
Il-1β	GCTTCAGGCAGGCAGTATCA	CTCTGCTTGTGAGGTGCTGA
Tnf-α	ATGAGCACAGAAAGCATGATC	TACAGGCTTGTCACTCGAATT
Il-22	GGTGTCTTGTGGCCTCCTATG	ATAGCCATCGGGACACCAGG
Il-23	CACCAGCGGGACATATGAATCT	CACTGGATACGGGGCACATT
Il-17A	TACCTCAACCGTTCCACGTC	TTTCCCTCCGCATTGACACA
Gapdh	CTGACTTCAACAGCGACACC	TAGCCAAATTCGTTGTCATACC
Sirt1	GAGCTGGGGTTTCTGTCTCC	CTGCAACCTGCTCCAAGGTA
Nrf2	AAAGCACAGCCAGCACATTC	TGGGATTCACGCATAGGAGC
Ho-1	GAACCCAGTCTATGCCCCAC	GGCGTGCAAGGGATGATTTC
Nqo1	AGTGGCATCCTGCGTTTCT	TCTCCTCCCAGACGGTTTC
Sod1	GCTTCTCGTCTTGCTCTCTCTG	TCTGCTCGAAGTGGATGGTT
Sod2	CCCAAAGGAGAGTTGCTGGA	TCTGTAAGCGACCTTGCTCC
D-loop	AATCTACCATCCTCCGTG	AATCTACCATCCTCCGTG
18S rRNA	AATCTACCATCCTCCGTG	CCTGCTGCCTTCCTTGGA
